# Comprehensive characterization of the alternative splicing landscape in head and neck squamous cell carcinoma reveals novel events associated with tumorigenesis and the immune microenvironment

**DOI:** 10.7150/thno.36585

**Published:** 2019-10-14

**Authors:** Zhi-Xuan Li, Zi-Qi Zheng, Zhuo-Hui Wei, Lu-Lu Zhang, Feng Li, Li Lin, Rui-Qi Liu, Xiao-Dan Huang, Jia-Wei Lv, Fo-Ping Chen, Xiao-Jun He, Jia-Li Guan, Jia Kou, Jun Ma, Guan-Qun Zhou, Ying Sun

**Affiliations:** 1 State Key Laboratory of Oncology in South China; Collaborative Innovation Center for Cancer Medicine; Guangdong Key Laboratory of Nasopharyngeal Carcinoma Diagnosis and Therapy, Sun Yat-sen University Cancer Center, Guangzhou 510060, P.R. China.; 2 School of Computer Science & Engineering, South China University of Technology, Guangzhou 510006, P.R. China.; 3 State Key Laboratory of Oncology in South China, Collaborative Innovation Center for Cancer Medicine; Department of Molecular Diagnostics, Sun Yat-sen University Cancer Center, Guangzhou 510060, P.R. China.

**Keywords:** head and neck squamous cell carcinoma, alternative splicing, genome-wide analysis, tumorigenesis, immune microenvironment.

## Abstract

Alternative splicing (AS) has emerged as a key event in tumor development and microenvironment formation. However, comprehensive analysis of AS and its clinical significance in head and neck squamous cell carcinoma (HNSC) is urgently required. **Methods:** Genome-wide profiling of AS events using RNA-Seq data from The Cancer Genome Atlas (TCGA) program was performed in a cohort of 464 patients with HNSC. Cancer-associated AS events (CASEs) were identified between paired HNSC and adjacent normal tissues and evaluated in functional enrichment analysis. Splicing networks and prognostic models were constructed using bioinformatics tools. Unsupervised clustering of the CASEs identified was conducted and associations with clinical, molecular and immune features were analyzed. **Results:** We detected a total of 32,309 AS events and identified 473 CASEs in HNSC; among these, 91 were validated in an independent cohort (n = 15). Functional protein domains were frequently altered, especially by CASEs affecting cancer drivers, such as PCSK5. CASE parent genes were significantly enriched in pathways related to HNSC and the tumor immune microenvironment, such as the viral carcinogenesis (FDR < 0.001), Human Papillomavirus infection (FDR < 0.001), chemokine (FDR < 0.001) and T cell receptor (FDR < 0.001) signaling pathways. CASEs enriched in immune-related pathways were closely associated with immune cell infiltration and cytolytic activity. AS regulatory networks suggested a significant association between splicing factor (SF) expression and CASEs and might be regulated by SF methylation. Eighteen CASEs were identified as independent prognostic factors for overall and disease-free survival. Unsupervised clustering analysis revealed distinct correlations between AS-based clusters and prognosis, molecular characteristics and immune features. Immunogenic features and immune subgroups cooperatively depict the immune features of AS-based clusters. **Conclusion:** This comprehensive genome-wide analysis of the AS landscape in HNSC revealed novel AS events related to carcinogenesis and immune microenvironment, with implications for prognosis and therapeutic responses.

## Introduction

Head and neck squamous cell carcinoma (HNSC) is a common, morbid and frequently lethal malignancy, with a global incidence of approximately 600,000 cases and accounting for around 380,000 deaths every year 1. Patients with HNSC are mostly diagnosed at a late stage, and often associated with poor prognosis. Despite advances in screening, diagnosis and multimodal treatments, approximately 50% of patients will die of the disease 2. Moreover, HNSC is characterized by clinical heterogeneity. Patients with aggressive disease are treated with cetuximab, an anti-EGFR antibody, but only about 13% of metastatic patients respond to this therapy 3. Recent studies have shown promising outcomes with anti-programmed cell death (PD)-1 therapy in advanced HNSC, although these agents benefit only a subset of patients 4-6. Accumulating evidence shows that multiple genomic alterations are required to unleash the malignant progression of HNSC 7. Hence, an improved understanding of the molecular mechanism underlying the pathogenesis of HNSC is urgently required. Furthermore, outcomes of clinical studies on therapeutics highlight the need to elucidate the inherent mechanistic disparities responsible for the variation in patient responses.

With the advent of next-generation sequencing, comprehensive genomic landscapes based on molecular characteristics of tumors such as somatic mutation and copy number variation have been studied extensively 7-12. Consequently, pathways frequently involved in the carcinogenesis, progression and metastasis of HNSC, such as p53, MAPK, PI3K and NF-κB signaling, have become a focus of research that has greatly improved our understanding of the genomic features of HNSC. More importantly, collective evidence shows that molecular patterns allow categorization of tumors into distinct subtypes associated with different clinicopathological features and prognosis 13, 14. However, these multi-omic analyses do not take into account alternative splicing (AS).

AS, which is one of the most important mechanisms of post-transcriptional regulation, is a regulated process by which RNA precursors are selectively spliced and joined, and can generate great biodiversity 15. Nevertheless, differential splicing of transcripts under pathological conditions may lead to structural and functional variation of proteins, some of which could be considered as potential drivers of tumorigenesis 16, 17. The unbalanced expression of splice isoforms or the failure to express the correct isoforms is considered another hallmark of cancer 18. Recently, a study characterized the AS landscape in the papillary thyroid carcinoma. Identification of differential AS events and further analysis of its potential regulatory mechanisms facilitated a transcriptome-wide understanding of PTC 19. More importantly, growing evidence demonstrates that AS plays an important role in immune microenvironment formation 20, 21. The AS alterations may not only affect immune cell infiltration but also regulate tumor-associated immune cytolytic activity. Additionally, cancer-specific AS changes have been recognized as important signatures to predict treatment efficacy in recent years. Daniel et al. discovered that patients with squamous carcinoma exhibiting a shifted splicing pattern toward EGFR isoform D respond to EGFR TKIs despite the absence of focal amplification or activating mutation in EGFR 22. Therefore, cancer-specific splice variants may not only serve as prognostic biomarkers, but also as potential therapeutic targets.

To the best of our knowledge, there is a scarcity of studies providing a comprehensive analysis of AS and its clinical significance in HNSC. In this study based on large-scale RNA sequencing data of TCGA HNSC samples, we conducted a systematic profiling of genome-wide AS events in HNSC and identified HNSC-related AS events. We further explored the potential biological function and underlying regulatory mechanisms of these events. In addition, integration of clinical information and RNA-Seq data provided an insight into prognostic value of AS events. Finally, we discerned distinct clusters of HNSC based on AS events and investigated the association between AS-based clusters and clinicopathological variables, tumor cell-intrinsic molecular characteristics, and immune features. Our analysis reveals a complex AS landscape consisting of both a continuous spectrum and discrete clusters across HNSC patients.

## Methods

### Data acquisition and curation process

Patients who met the following criteria were included in TCGA HNSC cohort: (1) histologically confirmed HNSC; (2) patients with RNA sequencing data; (3) patients with detailed clinicopathological and follow-up information [sex, age, TNM stage, HPV-status, overall survival (OS) and disease-free survival (DFS)]. Patients with metastasis were excluded because they were not universally represented. The corresponding RNA-Seq data were obtained from the TCGA data portal (https://tcga-data.nci.nih.gov/tcga/). RNA-Seq data of an independent cohort (ten tongue squamous cell carcinoma (TSCC) and five normal samples) was accessed from the European Nucleotide Archive (study accession: PRJEB14202). Data of differentially expressed AS events in colorectal cancer were referenced from a previous study 23. The copy number (CN) and methylation information of SF was retrieved from cBioPortal for Cancer Genomics database (http://www.cbioportal.org/) 24. Data of the four HNSC molecular subtypes and three HNSC immune molecular subgroups were accessed from Thorsson et al. 25 and Chen et al. 26, respectively. Immune and stromal scores were calculated to quantify the immune and stromal components in a tumor by using ESTIMATE algorithm 27. Data of immune cytolytic activity, mutational burden, and neoepitope abundance were referenced from a previous study 28.

### Generation of AS events profiling

RNA-Seq data were analyzed with SpliceSeq software 29 to generate the AS profiles for each patient as previously described 30-32. The Percent Spliced In (PSI) value is defined as the ratio of inclusion/exclusion normalized read counts as a percentage of the total (both inclusion and exclusion) normalized read counts for that event and was calculated for seven types of AS events. To generate a more reliable set of AS events, we implemented a series of stringent filters (percentage of samples with PSI values ≥75, average PSI value ≥0.05). We also removed the context-dependent AS events by measuring the significant association of the detected AS events with ESTIMATE scores. Spearman's rank correlation analysis was performed (|R| ≥ 0.4 and adjusted P < 0.05). Interactive sets among the seven types of AS were illustrated by UpSet plot created by UpSetR (version 1.3.3) 33. Circos plots were generated to visualize the detail of AS events and genes in chromosomes by Circlize (version 0.4.5) 34.

### Identification of cancer-associated AS events (CASE) and functional analysis

To identify cancer-associated AS events (CASEs) in HNSC, the PSI values of AS events were compared between HNSC and matched normal tissue. *P*-values were adjusted by Benjamini & Hochberg (BH) correction (|log2FC| ≥ 1, adjusted *P* < 0.05). Gene Ontology (GO) and Kyoto Encyclopedia of Genes and Genomes (KEGG) pathways enrichment analyses were conducted for the parent genes of identified CASEs (adjusted *P* < 0.05). Function enrichment analysis was performed using the “clusterProfiler” package (version 3.10.1) 35. Gene set enrichment analysis (GSEA) was performed to verify the differences in biological functions and pathways between tumor and normal tissues identified by clusterProfiler. Protein feature was analyzed by investigating whether there was a gain, loss or alteration of a protein feature (Pfam domains 36 or ProSite patterns 37). Spearman correlation analysis was performed to explore the association between CASEs related to immune pathways and microenvironment features. Immune cell infiltration was analyzed by CIBERSORT 38. Additionally, we mapped the parent genes of each CASE to coding proteins and built the interaction network using Search Tool for Retrieval of Interacting Genes/Proteins (STRING, version 11.0) 39, which was further visualized by Cytoscape (version 3.7) 40. Hub genes and modules were identified based on protein-protein interaction (PPI) network by cytohubba and MCODE in Cytoscape.

### Splicing Correlation network construction

Through screening published literature and relevant databases 41, 71 experimentally validated splicing factors (SFs) were identified belonging to two main families, Ser/Arg rich (SR) proteins and the heterogeneous nuclear ribonucleoproteins (hnRNPs), and other families such as CUGBP Elav-Like family (CELF), Fox and Nova families. Correlation analysis was performed between SF expression and PSI value of CASEs, and *P*-values were adjusted by BH correction (|R| ≥ 0.4, adjusted *P* < 0.05). The correlation plot was generated by Cytoscape (version 3.7). Correlation analysis was conducted to evaluate the association between SF methylation and SF mRNA expression. A two-sided *P*-value less than 0.05 was considered to indicate statistical significance.

### Survival analysis

HNSC patients were divided into two groups according to the median cutoff of each CASE. Univariate Cox regression analysis was performed to calculate hazard ratios (HRs) and 95% confidence interval (95% CI) of various CASEs in OS and DFS, respectively. Candidate prognostic AS events were then subjected to multivariate Cox regression analysis. The following clinical-relevant covariates were included in multivariate survival analysis: gender, age, TNM stage and HPV infection. Kaplan-Meier analysis with log-rank testing was applied to compare patients' survival in different groups. *P*-values were adjusted by BH correction.

### Evaluation of correlation with clinical, molecular and immune features

Based on CASEs, hierarchical consensus clustering was used to perform classification of TCGA HNSC cohort 14. To cluster in an unbiased and unsupervised manner, we used the “ConsensusClusterPlus” package 42. In our study, the clustering settings used were as follows: maxK= 8; cluster algorithm= pam; correlation method = Euclidean. Relative change in area under the cumulative distribution function (CDF) curve was used to determine the optimal number of clusters, k. To obtain a robust classification, the optimal numbers of clusters were further validated according to the total within sum of squares (WSS) and the gap statistics. The associations between clusters, clinicopathological variables (T stage, N stage, clinical stage and HPV-status), survival status (OS and DFS), molecular alteration (TP53 mutation, EGFR mutation/amplification), four HNSC molecular subtypes (classic, basal, mesenchymal and atypical) 10, 13, three HNSC immune molecular subgroups (non-Immune Class, Exhausted Immune Class and Active Immune Class) 26, and immune features (Immune Score, Stromal Score, immune cytolytic activity, mutational burden, and neoepitope abundance) were analyzed.

### Statistical analyses

All statistical analyses were performed in R (version 3.5.2), and P-value < 0.05 was considered statistically significant. Student's *t*-test and ANOVA test were utilized to compare continuous variables. Pearson's chi-square test and Fisher's exact test were employed for comparison of categorical clinicopathologic variables. Spearman's rank correlation analysis was used for non-normal distribution data. Pearson correlation was used for continuous variables that meet normal distribution.

## Results

### Overview of AS events in TCGA HNSC cohort

A total of 464 HNSC patients were identified and the baseline characteristics of these patients are summarized in **Supplementary Table S1**. During the median follow-up of 21.2 months (range, 1 to 210.8), 131 (28.2%) patients developed recurrence/progression and 203 (43.7%) patients died. The 3-year OS and DFS were 26.9% and 24.8%, respectively.

**Figure 1:**
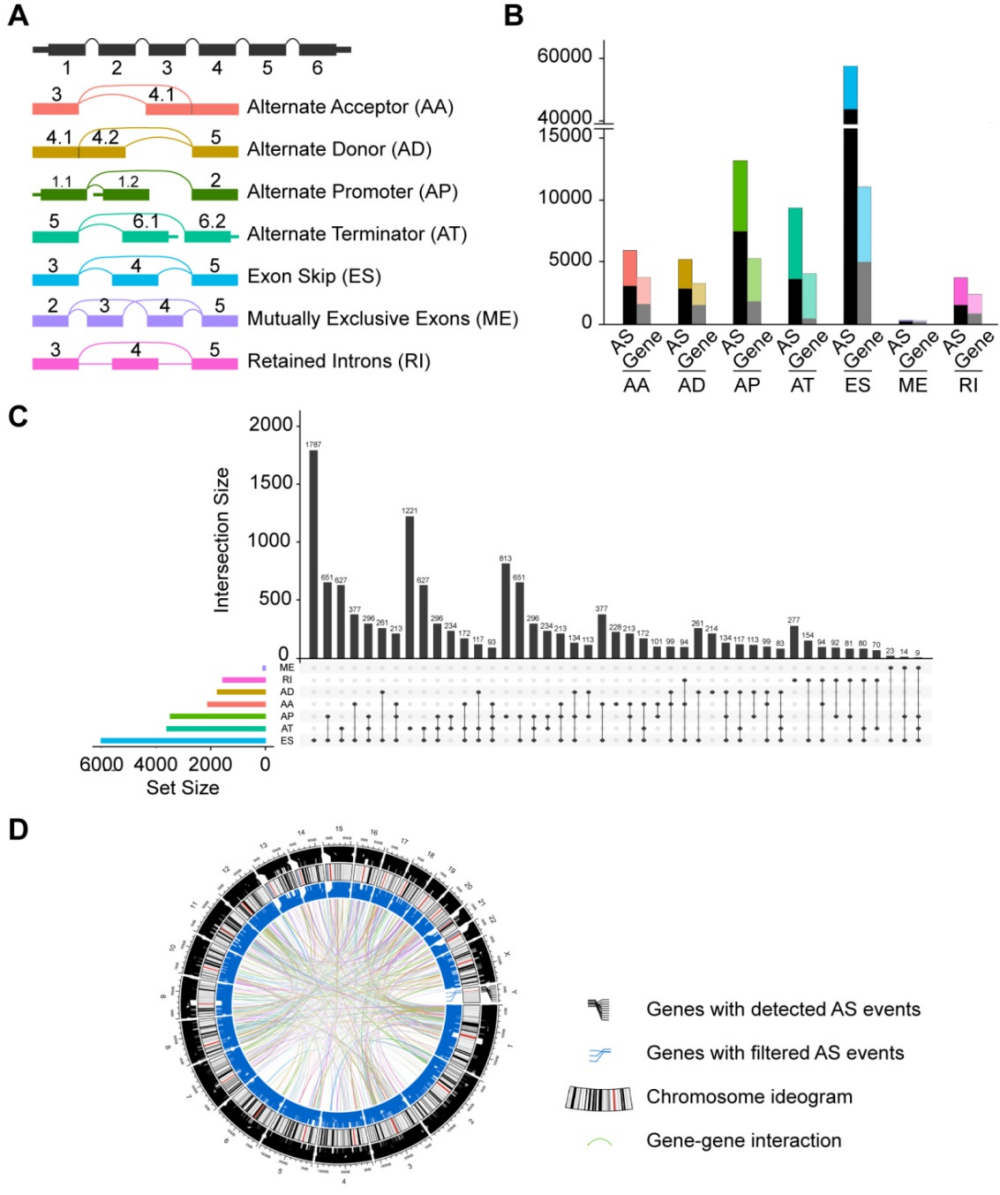
** Profiling of integrated AS events detected in HNSC. (A)** Schematic representation of seven modes of AS events: alternate acceptor site (AA), alternate donor site (AD), alternate promoter (AP), alternate terminator (AT), exon skipping (ES), retained intron (RI), and mutually exclusive exons (ME). **(B)** Number of AS events and their parent genes in the HNSC patients. Bar color represents the filtered AS events and their parent genes. Black bars represent the AS events and their parent genes filtered using stringent filters. **(C)** Interactive sets among seven modes of AS events (n = 32,309) shown in an UpSet plot.** (D)** Circos plot of the annotation of AS events and their parent genes in the chromosome. The outer circle is composed of the polyline that represents a detected AS event and links to the location of the parent gene in the chromosomes. The intermediate circle represents the chromosome ideogram. The inner circle consists if the polyline that represents a filtered AS events and links to the location of the parent gene in the chromosomes. The ribbons represent the potential interaction between the parent genes of cancer-associated AS events (CASEs).

The corresponding RNA-Seq data were used to establish integrated AS event profiling. We preliminarily detected 95,700 AS events from 13,862 genes, which accounted for approximately 67% of protein-coding human genes 43. These AS events were classified into seven splicing modes: alternate acceptor site (AA), alternate donor site (AD), alternate promoter (AP), alternate terminator (AT), exon skipping (ES), mutually exclusive exons (ME) and retained intron (RI), as illustrated in **Figure 1A**. Among these splicing modes, ES occurred most frequently (60.4%). It should be noted that a large proportion of the events were detected in only a small set of samples. In addition, certain isoforms were barely detected (PSI value < 0.05). To ensure high stringency, we screened the AS events with a series of filters (percentage of samples with PSI values ≥ 75, average PSI value ≥ 0.05). We also eliminated AS events with a significant association with stromal or immune cell content to specifically reflect cancer-associated AS alterations. Consequently, a total of 32,309 AS events from 9,844 genes were obtained (**Figure 1B**, **Supplementary Table S2**). After filtering, ES was still the most common mode (42.4%) followed by AT (17.4%) and AP (17.3%). Given the possibility of multiple splicing modes for a single gene, we created Upset plots to quantitatively analyze interactive sets of seven types of AS events. As shown in **Figure 1C**, a single gene could have up to four different splicing modes, and most genes had more than one AS events. Additionally, Circos plots were generated to gain a more intuitive visualization of AS event profiling of HNSC (**Figure 1D**).

### Identification of cancer-associated AS events in HNSC

**Figure 2:**
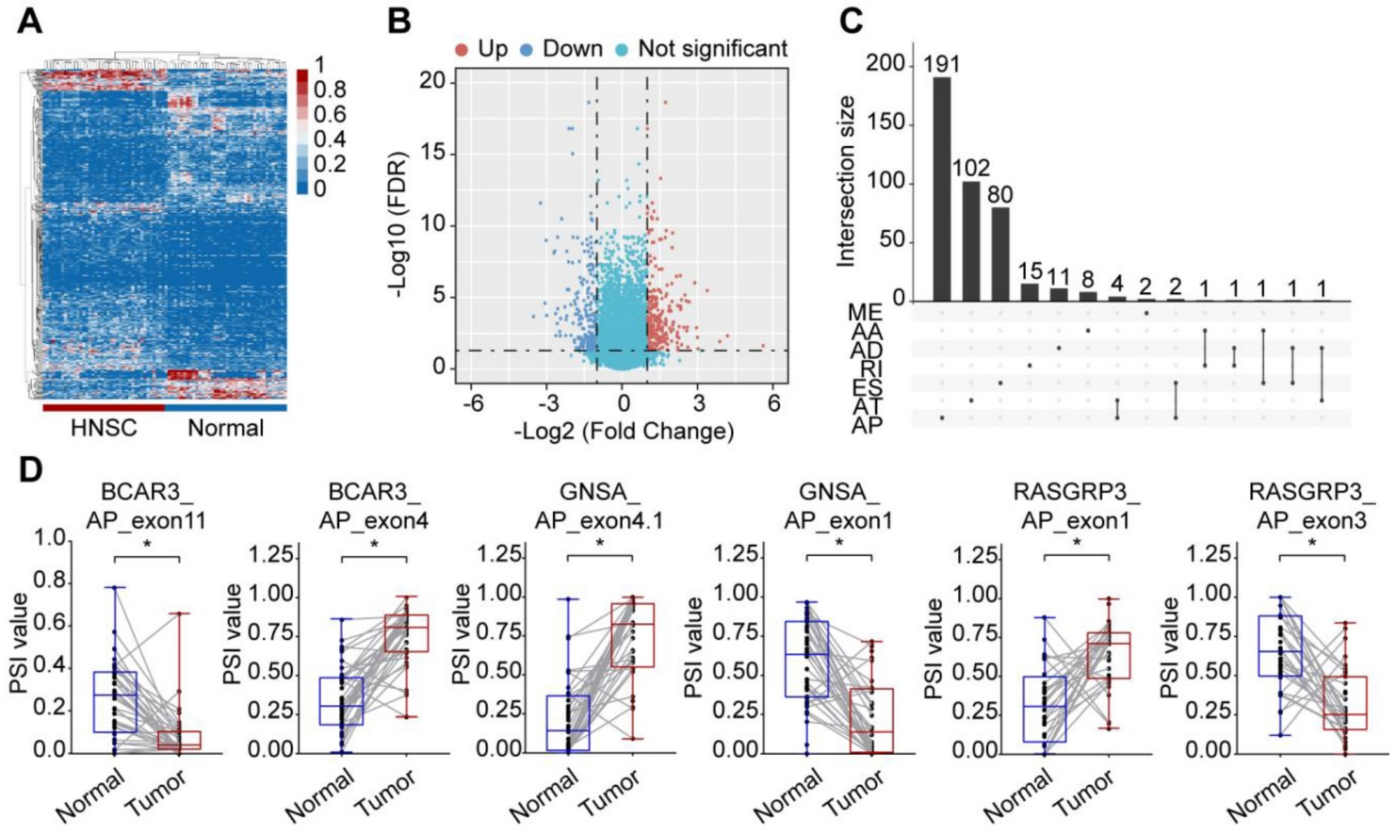
** Identification of CASE in HNSC. (A)** Heatmap of the CASEs between 40 pairs of HNSC and paracancerous tissues (|log2FC| ≥ 1, adjusted *P* < 0.05). **(B)** Volcano plot of CASEs identified in HNSC. The red and blue points in the plot represent upregulated and downregulated CASEs, respectively. **(C)** Different splicing modes of CASE (n = 473) shown in an UpSet plot. **(D)** The PSI value of representative CASEs showing the opposite preference between HNSC and adjacent normal tissues. Alternate acceptor site (AA), alternate donor site (AD), alternate promoter (AP), alternate terminator (AT), exon skipping (ES), mutually exclusive exons (ME), and retained intron (RI). Student's *t*-test was used. *: *P* < 0.05.

To identify the HNSC-specific AS events, we compared the PSI values between 40 paired tumor and adjacent normal tissues. After screening, a total of 473 cancer-associated alternative splicing events (CASEs) from 420 genes were identified (**Supplementary Table S3**). Out of 80 samples, only one normal sample was misclassified as tumor sample, an accuracy of 98.6%, which suggested that CASEs provided the ability to distinguish between tumor and normal tissue accurately (**Figure 2A** and** 2B**). The 473 CASEs in HNSC were further evaluated in an independent cohort (ten TSCC and five normal samples). After filtering, a total of 101 CASEs were detected, among which 91 CASEs were differentially expressed (**Supplementary Table S3**). Although a large number of ES events were detected in the HNSC cohort, a smaller proportion of ES events were identified as CASEs, while AP events accounted for 46.3% of CASEs (**Figure 2C**). There was an uneven distribution in the splicing patterns that suggested differed roles in cancer development. Moreover, several genes (such as BCAR3, GNAS, ISLR and RASGRP3) exhibited opposite patterns of AS events of a parent gene in tumor and normal tissues (**Figure 2D**).

Interestingly, among the 473 CASEs, 54 were differentially expressed in CRC as previously reported (**Supplementary Table S3**) 23. For example, AP of exon 1 in ISRL was significantly upregulated, whereas AP of exon 2 in ISRL was downregulated in both HNSC and CRC (**Supplementary Figure S1A**). We also found that ES events in TNC were generally more active in tumors (**Supplementary Figure S1B**). These events shed light on shared events in tumorigenesis. Given that aberrant AS might affect the expression of its parent RNA, especially when certain splicing modes (AP or AT) occurred, we evaluated the relationship between AS dysregulation and differential expression of mRNA. A total of 46 genes with CASEs were differentially expressed in HNSC, among which 39 (84.8%) were AP or AT events. We further studied the correlation between each AP/AT events and the corresponding gene expression in the HNSC cohort. Pearson's correlation analysis showed 40 of 45 (89%) AP/AT events were significantly correlated with parent gene expression, which indicated that CASE contributes to tumor development by dysregulating gene expression (**Supplementary Figure S1C-D, Supplementary Table S4**).

### Potential functions of CASEs

**Figure 3:**
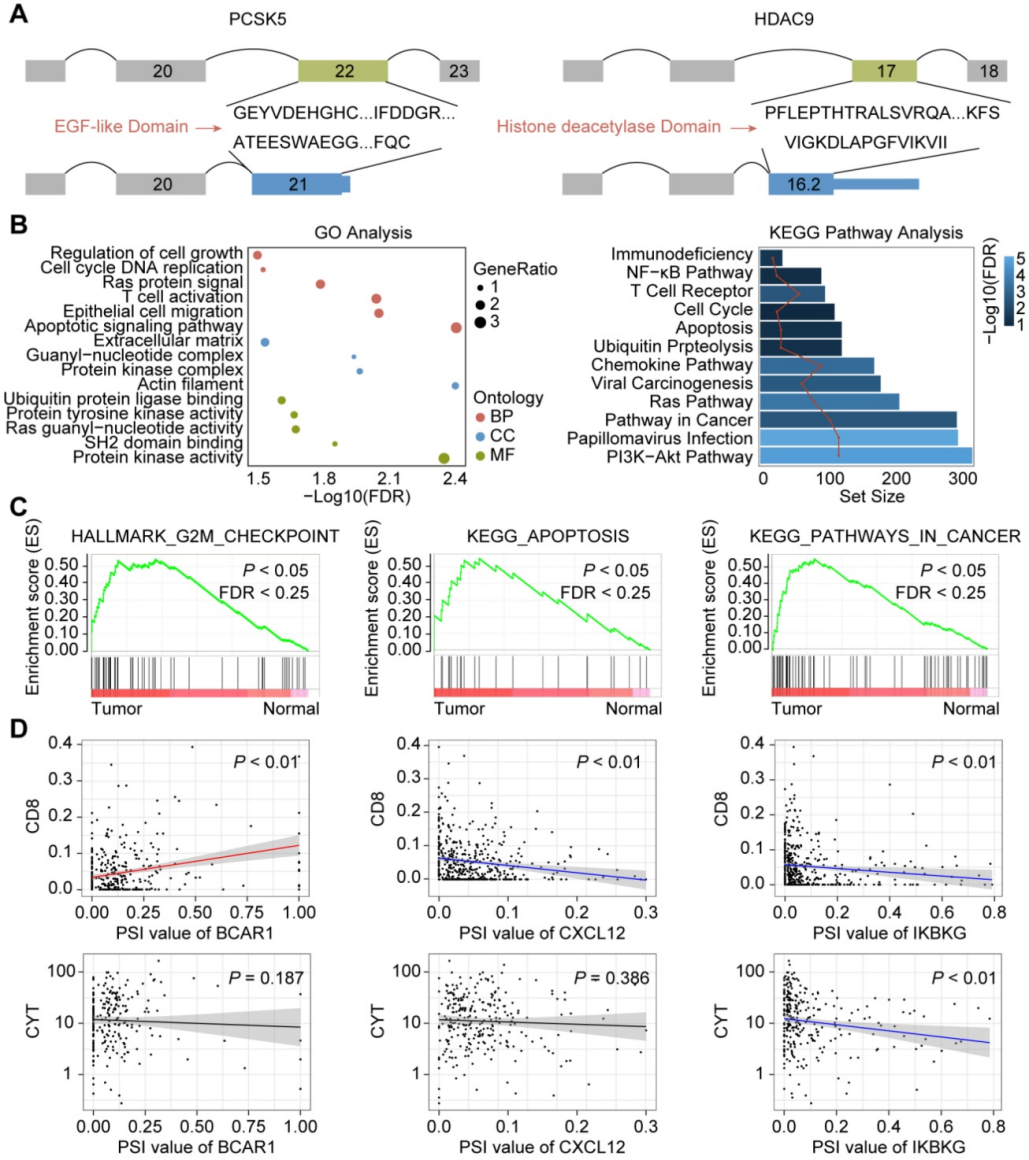
Potential biological function of CASEs. **(A)** Isoforms and proteins generated by CASEs of PCSK5 and HDAC9 affected the functional domains. **(B)** GO and KEGG analysis of CASEs. **(C)** GSEA analysis of CASEs. **(D)** Correlation analysis of specific CASEs and cytolytic activity/CD8+ T-cell infiltration. Adjusted *P* < 0.05.

Previous study suggested that alternative splicing (AS) events may affect similar domain families in cancer drivers and serve as potential drivers of cancer 44. To gain a better understanding of how CASEs may drive cancer development, we first investigated the CASEs affecting cancer driver genes. Among the CASEs, 26 events were identified to generate new isoforms in 22 cancer drivers, such as KRAS, SMARCA4 and FBXW7. Compared with canonical transcripts, transcripts generated from 18 CASEs tended to be shorter (**Supplementary Table S5**). In agreement with previous observations 17, all the cancer associated exon-cassette events were skipped, including ES events in KRAS and RHOT1, indicating protein feature losses or changes that was frequently observed in cancer progression. Inspired by this evidence, we studied the proteins encoded by the transcripts involved in AS events. Out of the 26 events, 22 caused altered protein sequence. As expected, annotated proteins encoded by CASEs tended to be shorter than proteins in normal isoforms. To determine the potential functional impact of CASEs, we explored the protein features (Pfam domains or Prosite patterns) they affected. To be note, domain families mediating interactions or involved in regulation of protein activity were involved (**Figure 3A, Supplementary Table S5**). AT event deprives PCSK5 of EGF-like domain (IPR000742), Tyrosine-protein kinase ephrin type A/B receptor-like domain (IPR011641) and Furin-like repeat (IPR006212), which may impair the cleavage-dependent maturation of furin. Recent study revealed that deficiency in PCSK5 caused inactive GDF11 precursor to accumulate intracellularly and promoted triple-negative mammary cancer metastasis 44. AT in HDAC9 results in the loss of histone deacetylase domain (IPR023801), which may lead to dysregulation of cell proliferation, apoptosis and cell cycle 45. ES in KRAS results in altered RAS domain family (PS51421) and small GTPase superfamily (Ras-type) and may further affect the signal transduction to downstream effector proteins. These findings suggested that CASEs may play important roles through dysregulating protein functions and provided clues for the exploration of tumorigenesis mechanisms.

Next, we analyzed the enrichment pathways of CASEs by biological function enrichment analysis. The results revealed that genes were enriched in GO categories closely related to HNSC development (**Figure 3B, Supplementary Table S6**), including regulation of apoptotic signaling pathway (FDR = 0.002), epithelial cell migration (FDR = 0.006), cell cycle DNA replication (FDR = 0.03) and regulation of cell growth (FDR = 0.03). We also noticed some biological function were more affected, such as Ras guanyl-nucleotide exchange factor activity and protein tyrosine kinase activity. Furthermore, some KEGG pathways associated with HNSC tumorigenesis were enriched (**Figure 3B, Supplementary Table S6**), such as viral carcinogenesis (FDR < 0.001), pathways in cancer (FDR < 0.001), apoptosis (FDR = 0.02). Intriguingly, immune-related pathways were also enriched, such as Human Papillomavirus infection (FDR < 0.001), chemokine signaling pathway (FDR < 0.001), T cell receptor signaling pathway (FDR < 0.001), and primary immunodeficiency (FDR = 0.010), which indicated that CASEs are also involved in HPV infection and immune microenvironment formation in HNSC patients. Consistent with these findings, Gene Set Enrichment Analysis (GSEA) revealed that AS events differentially expressed in HNSC were significantly enriched in cell cycle, viral carcinogenesis and immune-related pathways (**Figure 3C, Supplementary Figure S2**). Collectively, these findings suggested that CASEs may not only play important roles in tumorigenesis, but also in tumor immune environment.

Inspired by these findings, we further evaluated how CASEs may affect tumor microenvironment. Several CASEs were closely related to HPV infection and viral carcinogenesis, including PRKACB, CREB3L4, PXN, RELA and IKBKG. Correlation analysis revealed that higher expression of AP in IKBKG was associated with depletion of CD8 T cells and impaired cytolytic activity, which may be explained by NF-κB signaling pathways dysregulation (**Figure 3D**). We also observed that AT in IL1RL1, acting as a receptor to promote T cell activation, was associated with enhanced cytolytic activity 46. Moreover, CASEs involved in chemokine signaling pathways were also identified, including ES in BCAR1, AT in CXCL12, AP in ELMO1, AP in PTK2, AP in VAV1 and AT in GRK6. To be note, ES in BRAC1 and AT in CXCL12 were associated with recruitment of CD8 T cells but not with cytolytic activity (**Figure 3D**). Significant association between CASEs involved in chemokine signaling pathway and infiltration of different immune cells (except for CD8 T cells) were also identified. Thus, CASEs are closely associated with HPV-related carcinogenesis as well as tumor immune microenvironment.

**Figure 4:**
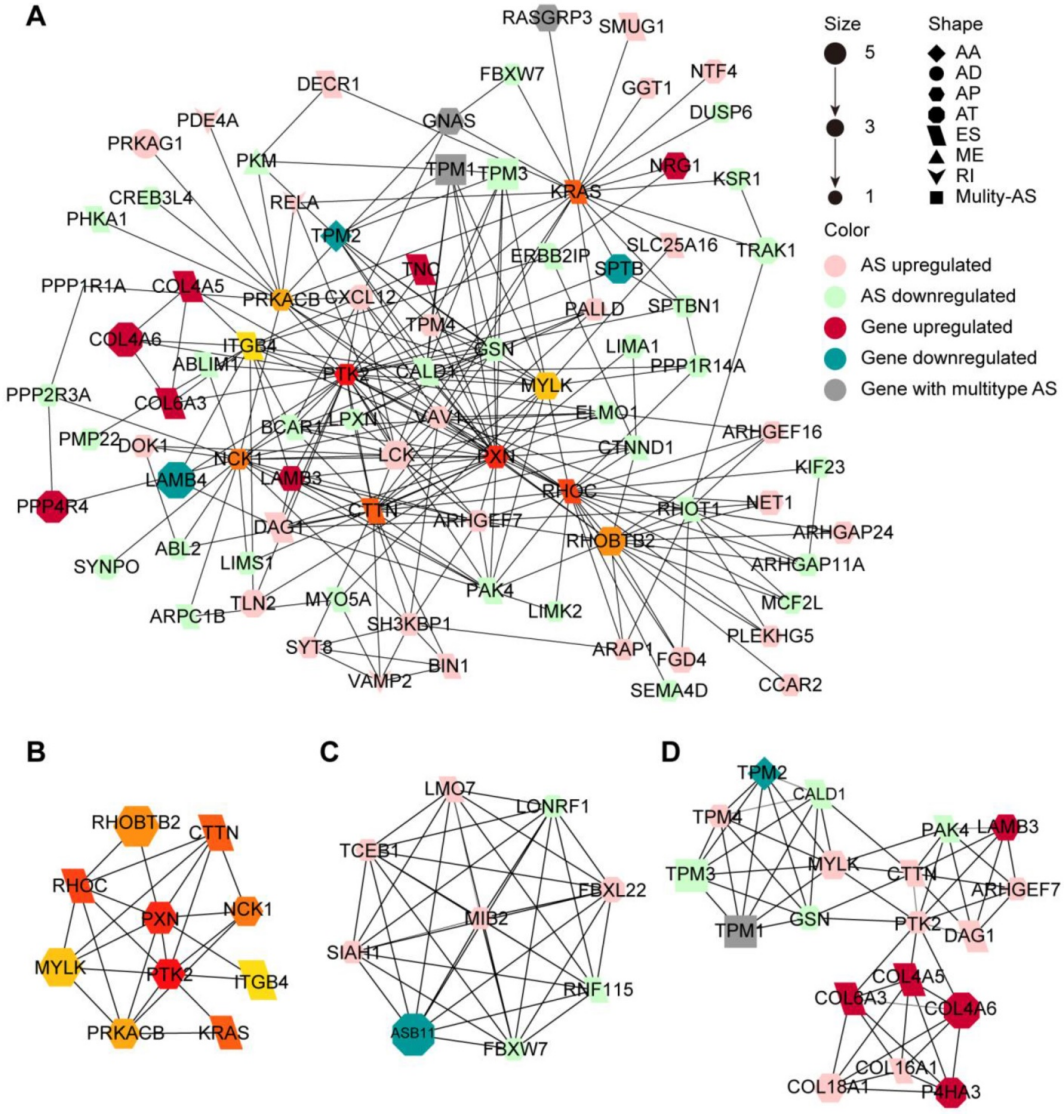
** Protein-protein interaction (PPI) network of CASEs. (A)** PPI network of CASEs generated by Cytoscape. Nodes represent genes with CASEs. The shape, color and size of nodes represent splicing modes, change patterns and |log2FC|, respectively. Edges represent the potential interactions between the corresponding protein. Alternate acceptor site (AA), alternate donor site (AD), alternate promoter (AP), alternate terminator (AT), exon skipping (ES), mutually exclusive exons (ME), and retained intron (RI). **(B)** Top 10 genes ranked by degree. **(C)** Module 1 was associated with biological processes of protein ubiquitination. **(D)** Module 2 was associated with cell adhesion and migration.

Since the proteins translated from alternatively spliced mRNAs vary in amino acid sequence and often have distinct biological function, we investigated the protein network of CASEs to provide an overview of the interactions in the normal state and uncover the potential influence of CASEs at the protein level (**Figure 4A**). We further identify the hub genes and modules based on the protein-protein interaction (PPI) network. The protein features of the hub genes were analyzed. Out of the ten hub genes, the functional domain families of eight genes were affected by the CASEs (**Figure 4B, Supplementary Table S5**). Both PTK2 and MYLK lost protein kinase domain suggesting that their protein phosphorylation activity may be lost when CASEs occurred. The kinase-dependent and kinase-independent functions of PTK2 moderate cell movement, invasion, survival and cancer stem cell self-renewal 47. MYLK regulates cytoskeleton by phosphorylating MLC and moderate epithelial-mesenchymal transition 48. We also identified two modules in the PPI network. Consistent with our findings in enrichment analysis, CASEs in the first module enriched in biological processes of protein ubiquitination (**Figure 4C**), suggesting that protein ubiquitination may be frequently affected by CASEs in HNSC. The other module comprises CASEs enriched in cell adhesion and migration (**Figure 4D**).

Taken together, our results suggested that HNSC-specific AS dysregulation plays a pivotal role in HNSC through promoting tumorigenesis and regulating immune microenvironment.

### Network of CASEs and splicing factors

**Figure 5:**
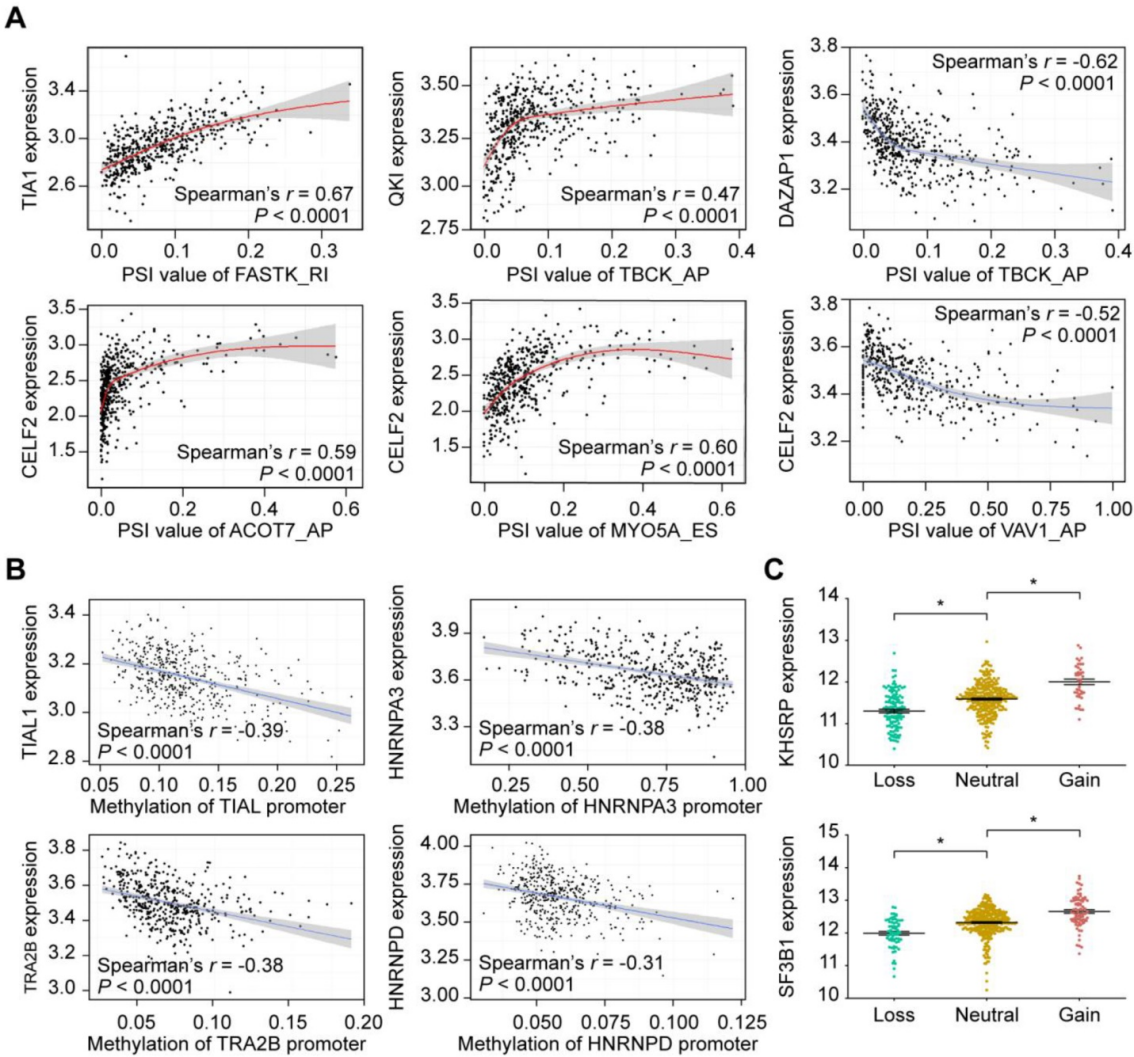
** Representative plots of regulatory splicing correlations in HNSC. (A)** Representative dot plots of correlations between expression of splicing factors (SF) and PSI values of AS events. **(B)** Representative dot plots of correlations between SFs promoter methylation and SFs expression. Data were analyzed using Spearman's rank correlation analysis (non-normal distribution) and Pearson's rank correlation analysis (normal distribution data). **(C)** Representative of SF expression between different copy number status. The expression value was calculated as log2(RSEM+1). The *P*-value was calculated by the Mann-Whitney test. All adjusted *P* < 0.05.

Splicing factors (SFs) are key regulators of AS events through selective inclusion of exons or removal of introns. SF alterations promote differential splicing patterns in tumors compared to normal tissues, resulting in the production of pro-tumorigenic isoforms 49. Thus, it is necessary to understand how CASE are regulated by SFs in HNSC. To gain insights into this issue, we analyzed the correlation between expression of 71 experimentally validated SFs and the PSI values of CASEs, and built a splicing regulatory network based on the significant correlations (**Supplementary Figure S3**). A total of 91 CASEs were associated with 40 SFs (**Supplementary Table S7**). Most SFs were significantly correlated with more than one AS event. In addition, one AS event could be regulated by up to 18 different SFs, which reflected the complex cooperative or competitive relationship between SFs and, in part, explained the diversity of splice isoforms created by only a few factors 50. **Figure 5A** shows some examples of highly correlated SFs and CSEAs.

To extend our findings to the potential mechanisms that contribute to altered expression of the splicing factors, we first investigated the relationship between SF promoter methylation and SF expression. Correlation analysis revealed that, among the 40 SFs, higher methylation level of 36 SF promoters was significantly associated with lower expression of the corresponding mRNA (**Supplementary Table S7**). As representatives, **Figure 5B** shows the relationship between the methylation levels of TIAL1, HNRNPA3, TRA2B and HNRNPD promoters and their expression levels. We further assessed the CNA events. Within the HNSC cohort, 85.6% cases possessed copy number (CN) loss and 94.0% contained CN gain events in at least one SF. CN loss of 29 SFs were associated with lower SF expression while CN gain of 30 SFs displayed significantly higher SF expression (**Figure 5C**,** Supplementary Table S7**). These findings suggested that genetic and epigenetic regulations may lead to changes in SF expression, and further regulates aberrant AS events.

### Prognostic value of CASEs in HNSC

Identification of biomarkers for early detection of disease and as potential therapeutic targets remains an important clinical issue. Previous studies have indicated that aberrant AS events occur in the early stages of cancer and are implicated as prognostic markers in many malignancies 51, 52. Therefore, we investigated the underlying relationship between CASEs and the prognosis of patients with HNSC.

**Figure 6:**
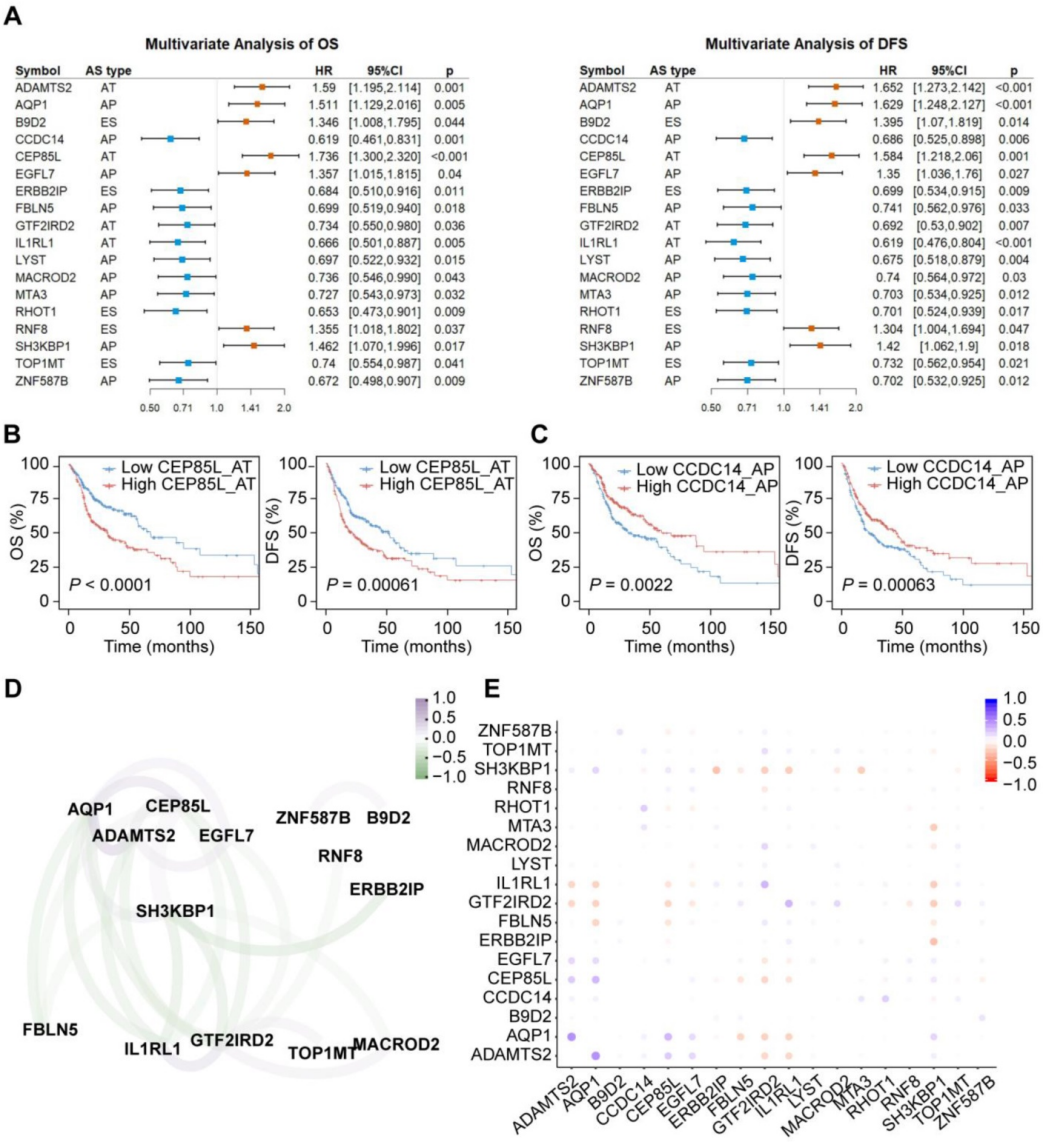
** The prognostic value of CASEs in HNSC. (A)** Forest plots of hazard ratios (HRs) for 18 CASEs associated with overall survival (OS) and disease-free survival (DFS). The red and blue boxes represent risk factors or protective factors, respectively. **(B-C)** Representative Kaplan-Meier curves for OS and DFS according to PSI value of AP in EGFL7 **(B)** and AT in IL1RL1 **(C)**. Multivariate Cox regression was used for data analysis. All adjusted *P* < 0.05. **(D)** The correlation network of survival-associated CASEs. The color of edge represents correlation coefficient. **(E)** The correlation plot of 18 survival-associated CASEs. The color of dot represents correlation coefficient.

For each CASE, we divided HNSC patients into two groups based on the median PSI value of each AS event. Univariate Cox regression analysis revealed that 59 and 53 AS events were significantly associated with OS and DFS, respectively. In subsequent multivariate regression analysis of these candidate events, 27 and 26 CASEs were found to be independent prognostic factors for OS and DFS, respectively. 18 AS events were simultaneously associated with OS and DFS (**Figure 6A**). AP in EGFL7 and AT in IL1RL1 are shown as representatives in **Figure 6B-C**. We also investigated whether these CASEs were independent of TP53 or EGFR mutation status and molecular subtypes (**Supplementary Table S8**). Among the 18 CASEs, there were 14, 15 and 9 CASEs independent of TP53 mutation, EGFR mutation and molecular subtypes, respectively, indicating that these CASEs are of important biological meaning.

Among the 18 CASEs, eleven events predicted improved prognosis while seven implicated worse prognosis. Similar to CASEs affecting cancer drivers, most CASEs associated with worse prognosis generated shorter transcripts and proteins. Unexpectedly, most CASEs associated with better prognosis generated longer transcripts and proteins (**Supplementary Table S5**). They expressed higher with an alternate promoter and lower with an exon skipping. Out of the eleven CASEs associated with better prognosis, seven events had a gain, loss or alteration in at least one protein feature (**Supplementary Table S5**). For example, TOP1MT was reported as an oncogene driving mitochondrial translation and carcinogenesis 53. Lower expression of exon skipping in TOP1MT resulted in an early termination of translation and therefore loss of DNA topoisomerase I feature (IPR001631) and topoisomerase I-related domains (IPR013499, IPR013500, IPR025834). Consequently, TOP1MT may lose its oncogenic function. Besides, AT in IL1RL1 may lead to gain of Toll/interleukin-1 receptor homology (TIR) domain and promote of T Cell Responses, which may explain the positive correlation between AT in IL1RL1 and cytolytic activity we observed above.

Since each tumor acquires multiple altered AS events, in order to examine if multi-CASE prognositic predictors can be collected, we assessed the co-occurrence and exclusivity of these survival-associated CASEs (**Figure 6D-E**). Correlation analysis revealed two highly-correlated groups of CASEs. One group, associated with improved survival, is centered on GTF2IRD2 accompanied with IL1RL1, TOP1MT and MACROD2. The other group mainly comprises an interaction among ADAMTS2, AQP1, CEP85L and EGFL7, which is associated with poor survival. Among these genes, several have been reported to play important roles in tumor development. Heterozygous and homozygous depletion of MACROD2 promotes chromosome instability and enhances intestinal tumorigenesis 54. ADAMTS2 is a metalloproteinase that inhibits angiogenesis and tumorigenesis 55. AQP1 regulates hydrostatic pressure-induced migration acceleration mediated by caveolin-1 and ERK1/2 signaling 56.

Collectively, our results directly suggested that CASEs are of not only biological importance, but also have potential clinical value.

### AS-based clusters significantly associated with prognosis, molecular characteristics and immune features

**Figure 7:**
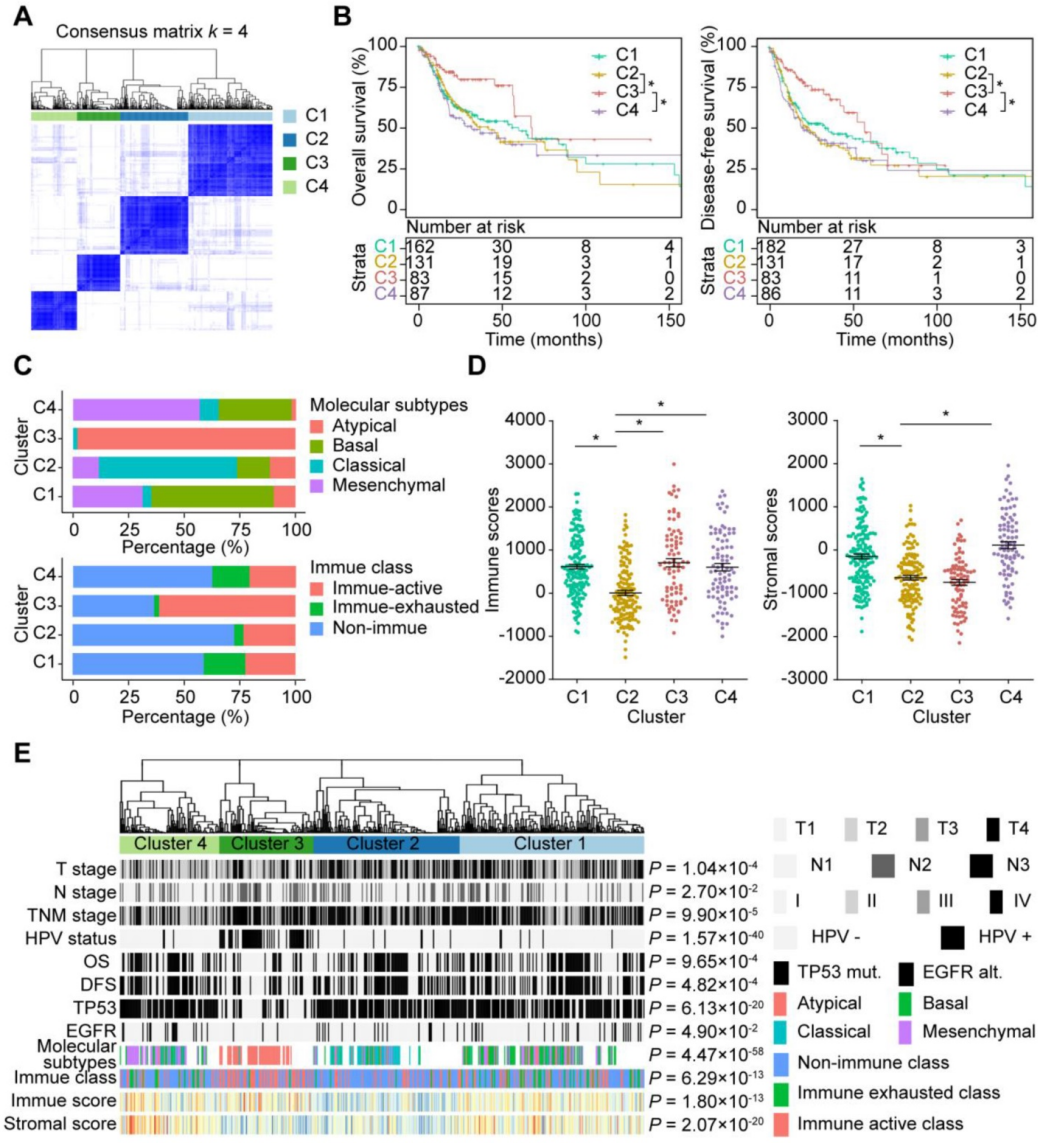
** AS-based clusters significantly associated with prognosis, molecular characteristics and immune microenvironment features. (A)** Consensus clustering analysis identification of four clusters (samples, n = 464). The white (consensus value = 0, samples never clustered together) and blue (consensus value = 1, samples always clustered together) heatmap display sample consensus. **(B)** Kaplan-Meier curves depicting survival probability over time for four AS-based clusters. Log-rank test was used for data analysis. **(C)** Bar plots of the relationship between HNSC molecular subtypes and HNSC immune class, and AS clusters. **(D)** Immune score and stromal score between AS-based clusters. Data were analyzed using ANOVA tests. **(E)** Heatmap of 473 CASEs ordered by clusters. The association with clinical, molecular and immune features was annotated. Chi-square test was used.

As shown by the AS profiling, AS events were remarkably heterogeneous among HNSC patients. Variation in CASE expression were predictive of different clinical outcomes for individual patients. To gain greater insight into the molecular heterogeneity of HNSCC, we performed unsupervised consensus analysis to explore whether AS presented discernable patterns. Based on the consensus matrix heatmap, the following distinct AS-based molecular clusters were identified (**Figure 7A, Supplementary Figure S4A**): C1 (n = 162, 34.9%), C2 (n = 131, 28.2%), C3 (n = 83, 17.9%), and C4 (n = 88, 19.0%).

To further clarify the clinical implications of the identified AS clusters, we first explored the relationships between clusters and clinicopathological characteristics. As shown in **Figure 7E**, TNM stage and survival status (OS and DFS) were not randomly distributed across different clusters (all *P* < 0.05). Moreover, Kaplan-Meier analysis of the association of clusters with prognosis revealed distinct patterns of survival (**Figure 7B**). Notably, C3 was associated with good prognosis in both OS and DFS, while C2 and C4 tended to carry poor prognosis. For two CASE groups with distinct prognostic values mentioned above, the group with better prognosis are highly expressed in C3, while the other group are highly expressed in C2.

To assess the correlation between AS and additional molecular characteristics, we further compared the four HNSC molecular subtypes (classic, basal, mesenchymal and atypical), TP53 mutations and EGFR alterations between clusters. It should be noted that highly significant similarities were observed between AS-based clustering and HNSC molecular subtypes (*P* = 4.47 × 10^-58^). Basal, classical, atypical and mesenchymal subtypes accounted for more than 50% in C1-C4, respectively (**Figure 7C**). Additionally, C3 tended to carry less frequent TP53 mutations and EGFR alterations (**Supplementary Figure S4B**). We also analyzed other genes that were frequently mutated in the TCGA HNSC cohort. Significant associations were observed between AS-based clusters and mutations in CDKN2A, FAT1, CASP8, PIK3CA, NOTCH1 and NSD1.

Given the potential function of CASEs in immune dysregulation shown in terms of functional enrichment, we investigated whether AS-based clusters present different immune patterns. Chi-square tests revealed that AS clusters were significantly associated with HPV (*P* = 1.57 × 10^-40^), which indicated that HPV-infected tumors (mostly in C3) display a specific AS pattern. Previous studies have shown that mutational loads and neoantigen abundance can reflect immunogenic features and predict responses to immunotherapy 57, 58. Our studies of total mutations and neoantigens abundance showed that C4 had both a significantly lower number of somatic mutations and neoantigens compared to other clusters. However, no significant difference was shown between HPV-related cluster C3 and C1-C2 (**Supplementary Figure S4C**). Furthermore, we investigated the differences in immune microenvironment between AS clusters. Immune and stromal scores were calculated based on the ESTIMATE algorithm to quantify the presence of stromal cells and the infiltration of immune cells in tumor samples. Interestingly, the HPV-related cluster C3 was associated with high immune and low stromal scores. We also noticed that C2 was associated with lower immune and stromal scores compared with C1 and C4 (**Figure 7D**). Additionally, our studies of the immune cell compositions and local immune cytolytic activity revealed that C3 showed enhanced cytolytic activity compared with other clusters (**Supplementary Figure S4B**), while C2 was associated with lower cytolytic activity. To comprehensively characterize the immune features in AS clusters and provide clues for immunotherapy, we assessed the correlation between AS clusters and three immune molecular subgroups in HNSC. The results suggested that AS clusters correlated with different immune status (**Figure 7C**,* P* = 6.29 × 10^-13^). A significantly higher frequency of patients with Active Immune Class was observed within C3 (60.2%). We also found that the non-Immune Class accounted for 73.3% of patients in the C2 cluster, while C1 and C4 were associated with a higher proportion of patients in the Exhausted Immune Class.

**Figure 8:**
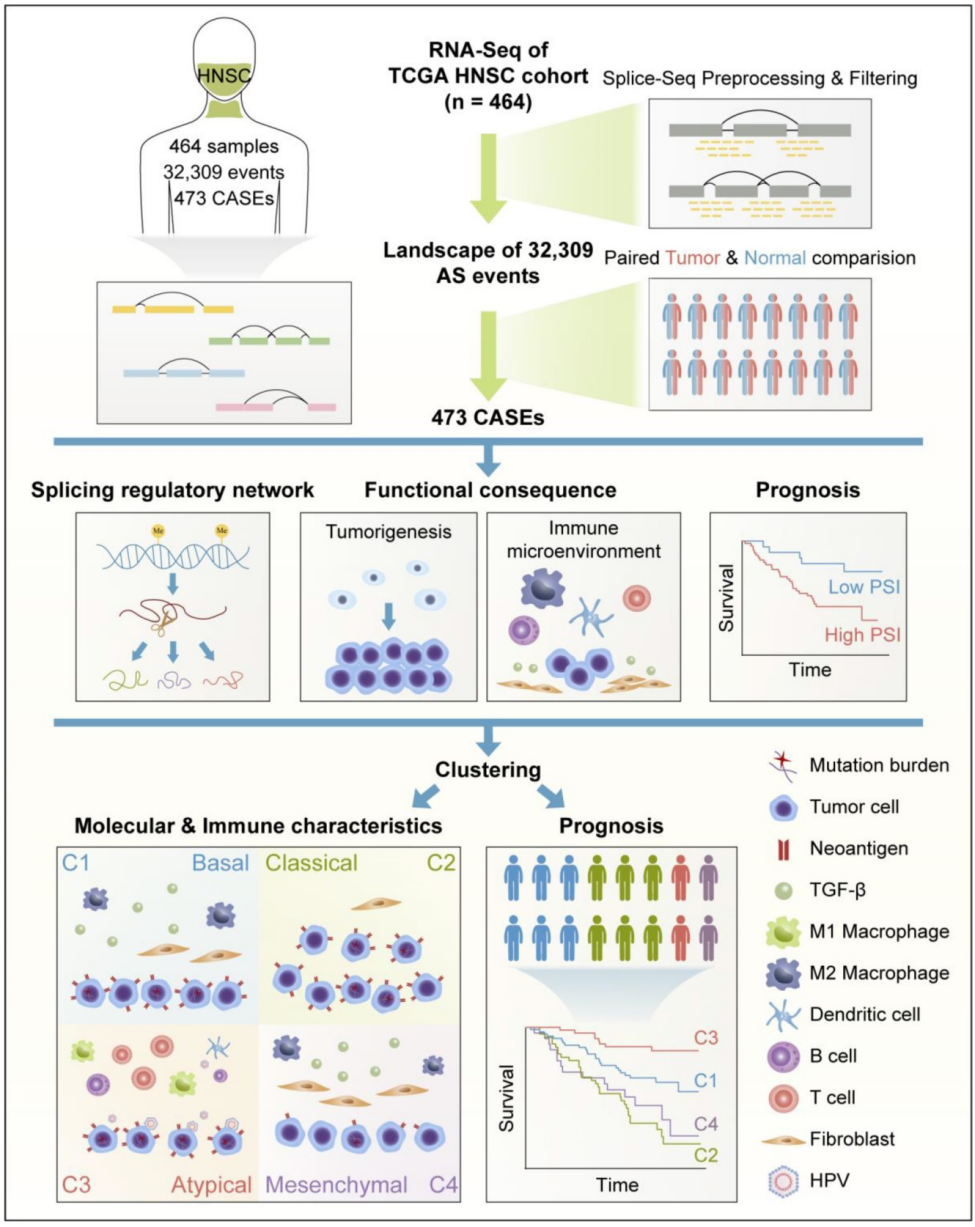
** Flowchart for comprehensive characterization of the AS landscape in HNSC.** RNA-Seq data of 464 HNSC patients were preprocessed and filtered by implementing rigorous filters (percentage of samples with PSI values ≥75, average PSI value ≥0.05). Comparison was conducted between paired HNSC and normal tissues. Based on cancer-associated AS events (CASEs), splicing regulatory network was built. Functional enrichment and survival analysis were performed. Clustering was conducted and associations with immunogenic and immune microenvironment features, molecular characteristics, and clinical outcomes were analyzed.

Collectively, these findings suggested that HNSC displayed distinct patterns of AS, and AS-based clusters serve not only as prognostic predictors, but also as potential indicators to define molecular targeted therapies and immunotherapeutic strategies for HNSC (**Figure 8**).

## Discussion

Traditional methods of genomic characterization have led to limited improvements in clinical outcomes for patients with HNSC, with high mortality rates and diverse therapeutic responses. The recent implication of AS in many biological functions has provided a new perspective for a better understanding of complex processes like cancer. However, the importance of individual AS events in HNSC has only been emphasized in a few cases. Here we systematically profiled the AS events in a large-scale HNSC cohort to elucidate the landscape of ASs in HNSC.

AS changes have been widely recognized in tumors for many years; however, systematic analysis is hampered by the current sequencing techniques and a dearth of advances in the analysis pipeline. Recently, accumulating evidence suggests that RNA-seq is a reliable and robust technique that can be employed in alternative splicing studies 59, 60. The results obtained using RNA-Seq with qPCR for differential expression analysis are generally consistent 60. Furthermore, further evidence indicates that AS events identified in RNA-Seq data play important roles in tumorigenesis 61, 62. Therefore, in the current study, RNA-Seq data was obtained and further analyzed by SpliceSeq 29, an integration tool which can detect low-frequency or complex events in an accurate manner. According to our results, the CASEs were significantly enriched in GO categories and KEGG pathways that were closely related to HNSC initiation or maintenance. More importantly, two AS events recently validated as functional in HNSC were also identified in our study 61, 63, which further confirms the suitability of RNA-Seq as a method to investigate AS changes in cancer. We also evaluated the 473 CASEs in an independent cohort. Among the 101 detected CASEs, 91 CASEs were validated, which suggested that CASEs identified in TCGA cohort may be ubiquitous in HNSC. Ryan et al. used exon microarrays to compare 44 tumor samples with 25 normal tissue samples and revealed 40 tumor-specific AS events 64. Despite applying a rigorous filter, a total of 473 CASEs from 420 genes were detected in the present study. Given that RNA sequencing enables the identification of novel AS events and provides better sequence depth and coverage than microarray assays, more cancer-related AS events may be identified with RNA-Seq data. Moreover, a direct comparison of paired tumor-normal tissue samples offers a better insight into hub events that participate in the biological processes of the tumor. However, adjacent normal tissues do not necessarily represent the origin of tumor cells, and expression changes might not be a necessary condition for spliced variants to be function 65. Therefore, more functional investigations will be required to validate the cancer specificity of AS variation. To be note, future studies incorporating long-read sequencing data are also in need to avoid artifacts of computational reconstruction and identify novel AS events.

Interestingly, HNSC has been reported to share some common cancer-specific AS events with CRC 23. As mentioned previously, opposite patterns of changes in two AP events in ISRL were observed in tumor and normal tissues, a phenomenon that is similarly observed in HNSC and CRC. Moreover, AP events in AQP1 and EGFL7 were upregulated in HNSC and CRC, and were associated with poor OS and DFS in HNSC, which indicated their important roles in carcinogenesis. We also observed that ES in TNC were generally active in tumors. TNC is a multi-modular matrix protein with a number of isoforms. Previous studies showed that TNC splicing may be involved in distinct cellular processes that drive embryogenesis and tumorigenesis 66. Collectively, these results shed light on the role of the shared AS events in tumorigenesis. Elucidation of the mechanisms underlying these events may provide valuable insights into potential therapeutic targets.

AS is known to be regulated by trans-acting SFs. Thus, we performed an integrated analysis of SFs and CASE expression to clarify the splicing pathway mechanism in HNSC. Our results revealed that aberrant expression of SFs was closely associated with CASE expression. Since protein-coding gene expression is subject to changes in promoter utilization that occur in carcinogenesis, we investigated the influence of SF promoter methylation on SF expression. A significant inverse correlation between promoter methylation and mRNA expression was identified for 36 SFs. Our results indicate that SF expression is regulated epigenetically and further contributes to the post-transcriptional AS changes in cancer.

Due to the potential significance of AS in tumor biology, its clinical relevance in malignancies has attracted increasing attention. In the present study, we conducted a systematic analysis of the prognostic value of CASEs and identified survival-associated AS events in HNSC. A total of 18 AS events were found to be independent prognostic factors of both OS and DFS. Among these AS events, several genes have been reported to play crucial roles in tumor biology. For example, ERBB2IP (ERBIN) has been reported to suppress RAS/RAF signaling and inhibit tumorigenesis in CRC 67. IL1RL1 inhibits proliferation and metastasis of CRC through modification of the tumor microenvironment 68. EGFL7 is a pro-angiogenic factor in glioblastoma and acute myeloid leukemia 69, 70, and a therapeutic target for ongoing clinical trials 71. Therefore, decoding the function and underlying mechanisms of these AS events may be significant for the development of novel therapeutic strategies.

To our knowledge, the current study is the first to conduct a systematic analysis of AS-based clustering of HNSC. Despite being a useful term for epidemiologic purposes, HNSC represents a cluster of tumors originating from the oral cavity, oropharynx, and laryngeal sites. Due to its biological heterogeneity, the use of anatomic sites, TNM stage and HPV-status to stratify high-risk patients and tailor treatment options has limited clinical utility. Recent genome-wide studies showed that HNSC can be clustered into molecular subgroups based on distinct patterns of gene expression or immune spectra, which provides clues for targeted therapy and immunotherapy 10, 14, 72. In the present study, four AS-based clusters were identified. TNM stage, HPV-status, TP53 mutation, EGFR mutation/amplification and survival status were unevenly distributed among AS clusters. Intriguingly, AS-based clusters (C1-C4) resembled molecular subgroups defined by patterns of gene expression (classical, basal, atypical and mesenchymal). This result demonstrated close associations between subgroups defined by AS and gene expression.

An interesting finding of the present study was that AS clusters presented distinct immune features. Previously, a multi-omic analysis of the molecular features in HNSC demonstrated strong differences between HPV-positive and HPV-negative tumors 10. Our analysis suggested that HPV-infected tumors present a specific AS pattern, which may partially explain the difference in immune microenvironment between clusters. Recently, Chen et al. identified three novel immune molecular subgroups based on immune microenvironment in HNSC, which might aid the identification of ideal candidates for treatment and the ability to tailor optimal immunotherapeutic strategies 26. In the immune molecular subgroups of HNSC, Active Immune Class is associated with an enriched proinflammatory M1 macrophage signature and abundant tumor-infiltrating lymphocytes, which reflect a better response to immunotherapy. Among the four AS clusters, HPV-related cluster C3 was associated with enriched immune infiltration, enhanced cytolytic activity and a high proportion of patients in the Active Immune Class. Based on this interesting phenomenon, we hypothesized that HPV infection may impact the development of anti-tumor immune responses and the presence or composition of tumor-associated immune cells by regulating AS events. Except for HPV-related C3, we also observed distinct immune microenvironment and immunogenic features in other three clusters (C1, C2 and C4). C2 was characterized by low immune infiltration, impaired cytolytic activity and a high frequency of patients in the non-Immune Class, while C1 and C4 were both associated with enriched stromal cells and a high proportion of patients in the Exhausted Immune Class. We also noticed that C4 had a significantly lower number of somatic mutations and neoantigens compared to C1.

In summary, implementation of rigorous criteria ensured the identification of ubiquitous AS events related to HNSC. Our function enrichment analysis indicates that the 473 CASEs identified in this study may play important roles in HNSC tumorigenesis. SF correlation networks further clarify the underlying mechanism of the splicing pathway. In addition, survival-associated AS events may not only be valuable in deciphering the mechanisms of AS in oncogenesis, but also serve as potential clinical biomarkers and therapeutic targets. More importantly, a comprehensive clustering analysis of HNSC based on AS revealed the intrinsic relevance of molecular alterations and immune features, and indicates the value of this information in predicting clinical outcomes in patients with HNSC.
