# Supplementary material for: Comprehensive characterization of the alternative splicing landscape in head and neck squamous cell carcinoma reveals novel events associated with tumorigenesis and the immune microenvironment
Source: Theranostics. 2019 Oct 14;9(25):7648–65. doi: 10.7150/thno.36585 (PMC6831462; doi:10.7150/thno.36585)
Supplement: Supplementary file 1 — Supplementary figures. [file thnov09p7648s1.pdf]

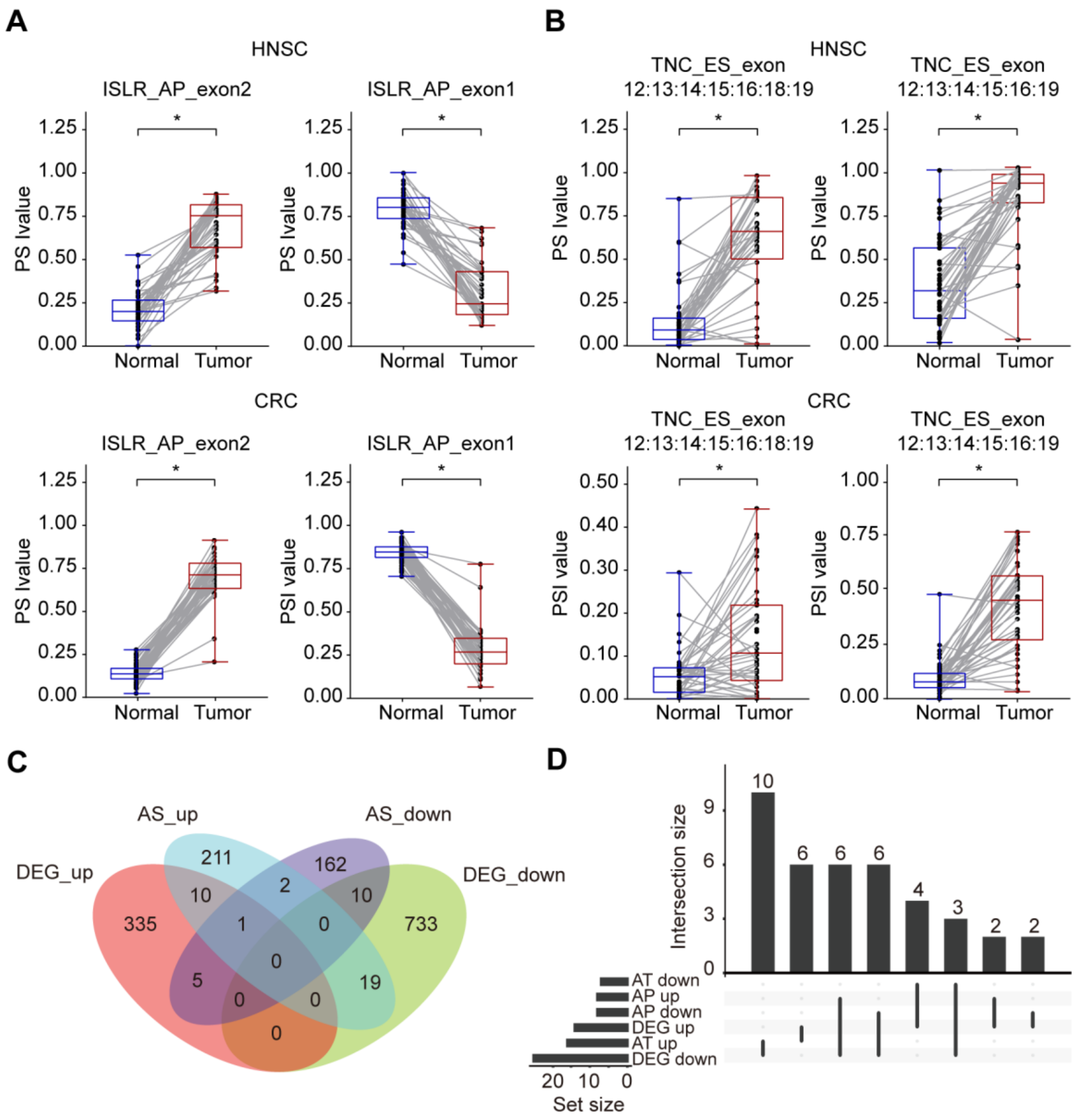

Supplementary Figure S1. (A-B) The characteristics of CASEs. **(A)** AP of exon1 and exon2 in ISLR show an opposite preference between tumor and normal tissue. **(B)** Two ES events in TNC shown to be more active in tumor compared with normal tissue. *: $P < 0.05$ **(C)** Venn diagram of CASEs and differentially expressed genes (DEG). **(D)** Interactive sets of AP/AT events and DEG displayed in an UpSet plot.

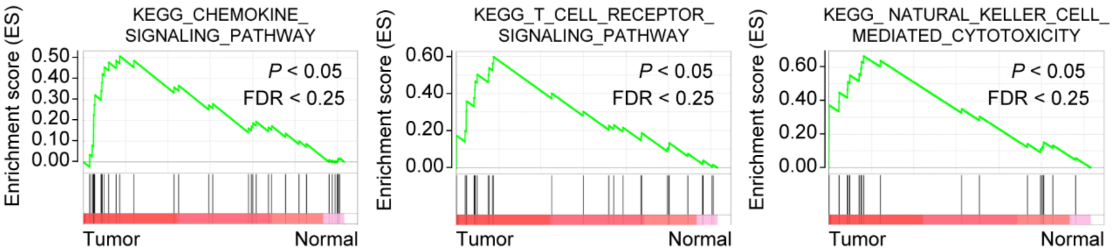

Supplementary Figure S2. The GSEA analysis of CASEs. The CASEs were enriched in immune-related pathways by GSEA.

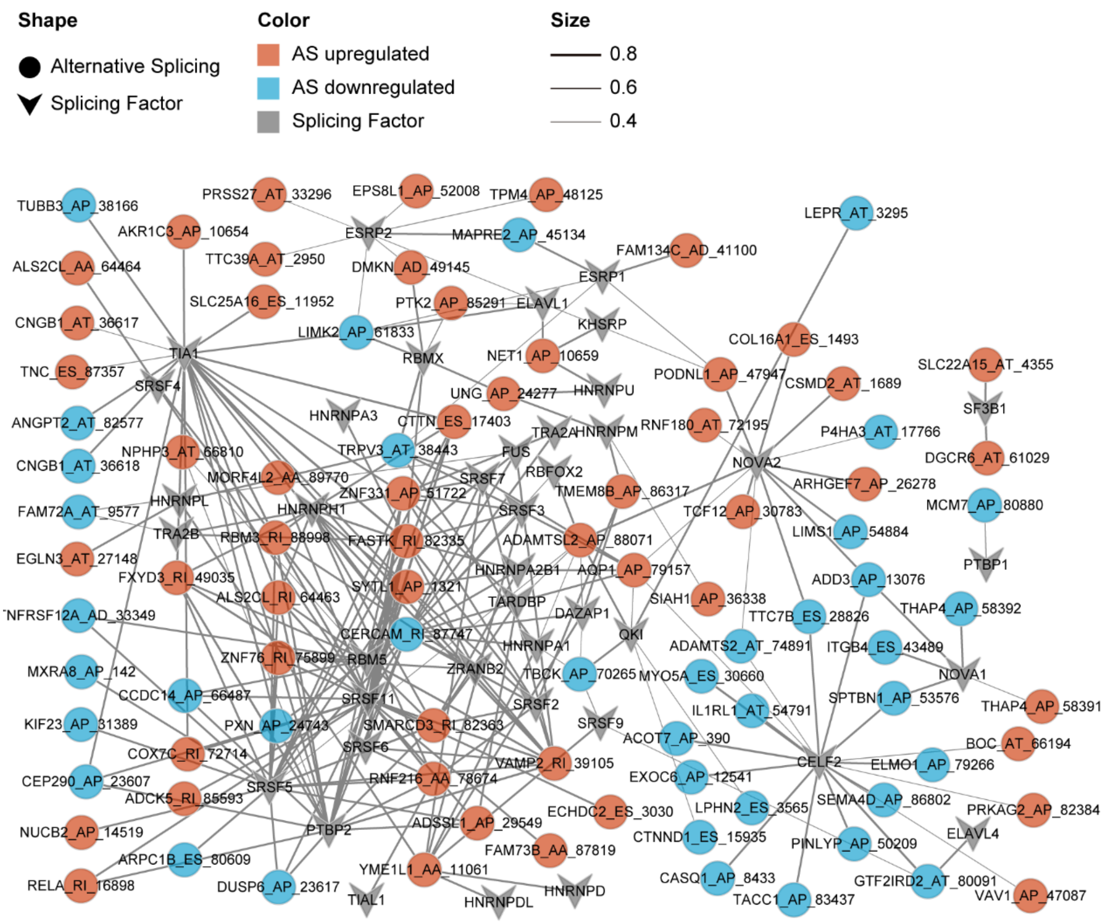

Supplementary Figure S3. Correlation network of splicing factors and CASEs in HNSC. The splicing correlation network was built based on significant correlations between the expression of 71 splicing factors and the PSI values of CASEs. A node represents an AS event or a splicing factor, which is distinguished by shape of the node. The color and size of node represent change pattern (upregulated or downregulated) and |log2FC|, respectively. The width of each edge represents the value of correlation coefficient. Alternate acceptor site (AA), alternate donor site (AD), alternate promoter (AP), alternate terminator (AT), exon skipping (ES), mutually exclusive exons (ME), and retained intron (RI).

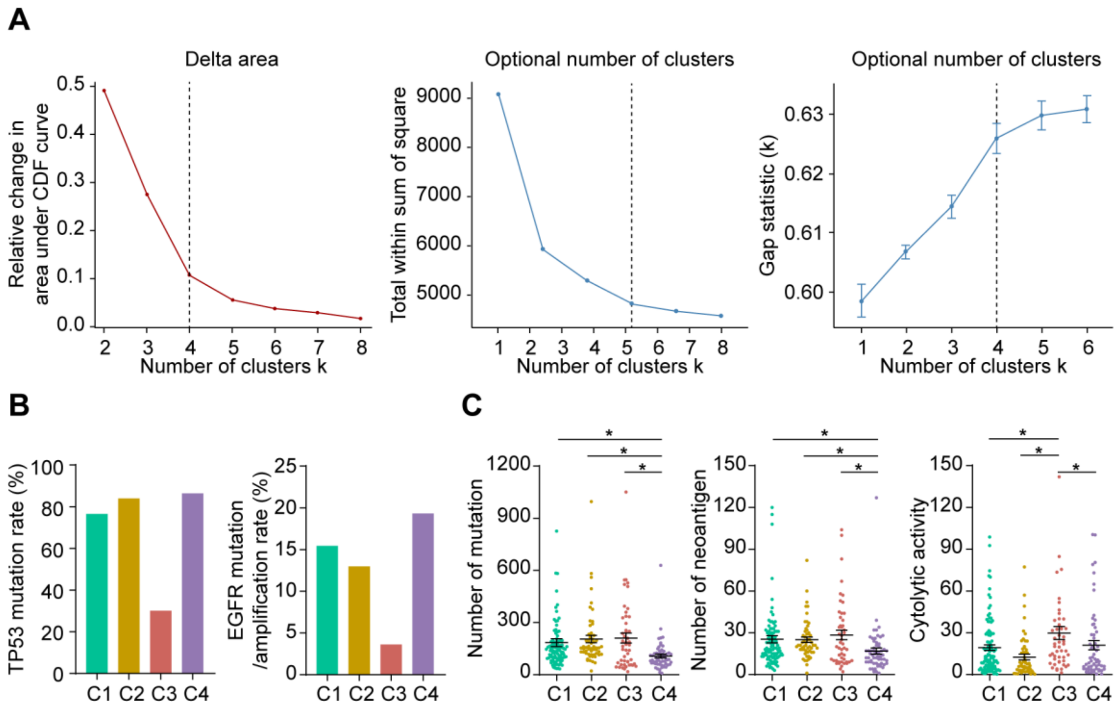

Supplementary Figure S4. Hierarchical clustering and the association between AS-based clusters and molecular/immune features. (A) Relative change in area under the CDF curve was used to identify optimal cluster number k (k = 2 to 8). Total within sum of square and gap statistic analysis was used to further validated the optimal cluster number k (k = 2 to 6). **(B)** Bar plots of the percentages of TP53 mutation and EGFR mutation/amplification among AS-based clusters. **(C)** Mutation loads, neoantigen abundance, and immune cytolytic activity in AS-based clusters. Data analyzed using ANOVA tests. *: $P < 0.05$
